# Technologies for strengthening immunization coverage in India: a systematic review

**DOI:** 10.1016/j.lansea.2023.100251

**Published:** 2023-08-08

**Authors:** Nonita Dudeja, Tila Khan, Deepak Thomas Varughese, Sebin George Abraham, Marilyn Mary Ninan, Christie Leya Prasad, Rajiv Sarkar, Gagandeep Kang

**Affiliations:** a Centre for Health Research and Development, Society for Applied Studies, New Delhi, India; b School of Medical Science & Technology, Indian Institute of Technology Kharagpur, West Bengal, India; c Department of Community Medicine, Believers Church Medical College, Thiruvalla, Kerala, India; d Department of Community Health, Christian Medical College, Vellore, Tamil Nadu, India; e Department of Clinical Microbiology, Christian Medical College, Vellore, Tamil Nadu, India; f Christian Hospital Bissamcuttack, Rayagada District, Odisha, India; g Indian Institute of Public Health Shillong, Shillong, Meghalaya, India; h Division of Gastrointestinal Sciences, Christian Medical College, Vellore, Tamil Nadu, India

**Keywords:** Technology, Immunization, India, Systematic review, Coverage

## Abstract

**Background:**

Immunization coverage varies across India in different settings, geographic areas and populations. Technologies for improving immunization access can reduce disparities in coverage. This systematic review, which follows PRISMA guidelines, aims to examine the technologies for strengthening immunization coverage in India.

**Methods:**

Studies published between January 1, 2011 and July 31, 2021 were searched in Medline (through PubMed), Cochrane Library and Google Scholar. All observational and experimental studies, except qualitative studies, were included. Studies published in the English language and related to technologies for strengthening immunization, conducted on children, pregnant women, adults, elderly, healthcare personnel, caregivers and vulnerable populations across all Indian settings were included. Non-English articles, protocols, commentaries, letters, abstracts, correspondence, opinion articles, modelling, narrative and systematic reviews were excluded. Two reviewers screened studies independently, extracted data in a standardized sheet and appraised the study quality using the Mixed Methods Appraisal Tool. The primary outcome was technologies that improved immunization coverage. The protocol is registered with OSF (https://osf.io/r42gm).

**Findings:**

6592 titles and abstracts were screened, and data extracted from 23 India-specific studies. Quality of 22/23 studies was average or above. Technologies identified included reminder systems, capacity building, community engagement and wearable technologies. Automated incentivised mobile phone reminders, immunization due-list, computerized data tracking, community mobilization and campaigns improved vaccine coverage, although effectiveness of some varied viz., reminder systems, and across states. Newer technologies included the Jyotigram Yojana, Digital Near-field Communication Pendants, “Reaching Every District” Programme and the “My Village My Home” tool.

**Interpretation:**

Technologies for improving immunization systems, capacity building and community engagement were effective. Newer technologies on vaccine delivery, mapping and cold chain logistics were not evaluated in India or were ineffective. There were limited studies in populations other than children and pregnant women. Future work is needed to evaluate the effectiveness of identified technologies across diverse settings.

**Funding:**

No funding was received for preparing this manuscript.

**Research in context**

**Evidence before this study**

Evidence on technologies or interventions aimed at improving immunization coverage in India have mainly focused on specific age groups, populations, vaccines, settings, or a specific technology. To our knowledge, so far, no systematic review has focused on technologies used in the Indian context that covers all populations and settings. In this systematic review, we searched three databases, Medline (through PubMed), Cochrane Library and Google Scholar, for published evidence related to technologies that influence immunization coverage in the Indian setting. All observational and experimental studies conducted on all populations across India, published in English-language, between January 1, 2011 and July 31, 2021, were included. Qualitative studies, study protocols, commentaries, letters, correspondence, opinion articles, narrative reviews, systematic reviews, blogs and newspaper articles were excluded. We used the MeSH and Title abstract terms for vaccination and immunization, patient acceptance and vaccine uptake, and for types of technologies. The methodological quality of included studies was appraised using the mixed methods appraisal tool (MMAT).

**Added value of this study**

This review collates all available evidence on technologies for strengthening immunization coverage in India and covers all age groups, population types (children, adults, elderly, pregnant and lactating women, health care professionals, people with comorbidities and marginal populations) and settings. We summarise the evidence by categorising different technologies under reminder systems, capacity-building initiatives, community engagement initiatives, intersectoral coordination, wearable technology, regulation and monitoring and advocacy. Some technologies were established and successful such as immunization campaigns (including the polio campaign), Mission Indradhanush and Measles-Rubella campaign, while some were relatively new such as digital near-field communication pendants. Studies have also used multi-pronged strategies for strengthening overall immunization programs. Reminder strategies showed variable results. Whether one technology is better than the other remains uncertain. Future studies are required to ascertain the effectiveness and acceptability of single or multiple technologies for overall program improvement in different contexts, populations and geographic locations.

**Implications of all the available evidence**

Our study identifies multiple technologies that have been successful in the Indian context. These technologies need to be further explored and assessed for possible replication in low-performing districts, different populations, particularly vulnerable or vaccine-hesitant and in hard-to-reach areas. This study will provide an evidence base for informing policy decisions on improving immunization programs and strengthening the healthcare system in India. At the same time, this study also highlights the lack of studies on impact of technologies on immunization of populations other than children and pregnant women. There are certain well-established technologies in other countries that have not been studied in India. Future research needs to be directed at studying newer technologies for improving immunization and also assessing the impact of existing successful technologies across other districts of the country.

## Introduction

Immunization is a simple public health intervention that reduces the burden of many vaccine preventable infectious diseases and healthcare expenditure.^1^ Through immunization the global mortality of children under 5 years from vaccine-preventable diseases reduced from 5.1 to 1.8 million through 1990–2017.^2^ Beyond infancy and childhood, vaccines save the lives of adolescents, adults, pregnant women, high-risk population (healthcare personnel, immunocompromised individuals, occupationally exposed individuals, migrants, populations living in remote and conflict settings) and the elderly, thus laying the foundations of healthy and productive populations.^3^ Immunization of adults through catch-up and booster vaccinations provides longstanding protection, thus facilitating healthy ageing and well-being.^4^

The global immunization coverage for all ages dropped from 86% in 2019 to 81% in 2021.^5^ While the COVID-19 pandemic has been strongly instrumental in this decline, the importance of raising global coverage to more than 90% remains a priority. Excluding COVID-19 vaccine introductions, only 25 vaccines were introduced globally in 2021. Even though this is an increase from 17 introductions in 2020, when compared to number of vaccines introduced in any year, in the past two decades prior to 2020, this number is quite low. As per the World Health Organization (WHO) data on global immunization coverage, 18.2 million infants missed receiving the first dose of childhood diphtheria, tetanus, pertussis (DTP) vaccine series in 2021, suggesting the impact of COVID-19 on access to immunization and other health services.^5^ More than 60% of unvaccinated or partially vaccinated children in 2021 were from 10 countries viz., India, Nigeria, Indonesia, Ethiopia, Philippines, Democratic Republic of the Congo, Brazil, Pakistan, Angola, and Myanmar.^5^ More recently, the introduction of COVID-19 vaccines too was highly variable across countries in 2021, with cumulative number of doses administered per 100 people ranging from 118 in Israel, to less than 0.1 in countries that had just begun vaccination such as Namibia, Mali and Brunei.^6^ India faces challenges of underfunded and overstretched health care system, weak surveillance and immunization infrastructure, reduced access to healthcare, lack of awareness and socio-cultural barriers to healthcare utilization.^7^ Strengthening immunization programs is essential to meet the regional and global disease elimination targets and to achieve the Sustainable Development Goal 3.

A concerted effort is needed to use innovative technologies, digital solutions and other approaches to strengthen the immunization programs so that no person is left behind as part of universal health coverage (UHC).^8^ Different technologies have been used as interventions to address inequitable access, suboptimal uptake and vaccine hesitancy.^9^ Technology offers significant potential to improve vaccine coverage and could be targeted for health system strengthening, regulation, program monitoring, evaluation, logistics management, capacity building, information and communication etc. The applicability and availability of technology types could vary with the geographical region, target population, context and setting.

Even though narrative and systematic reviews on effectiveness of technologies for immunization have been conducted earlier, they were limited to either of the World Bank income groups, or focused on specific populations,^10^, ^11^, ^12^, ^13^, ^14^, ^15^ target groups,^14^, ^15^, ^16^ vaccines,^16^^,^^17^ or technology type/intervention.^9^, ^10^, ^11^, ^12^, ^13^^,^^18^ A comprehensive real-world evaluation of the existing technologies in India, across all populations and settings, are needed for a better understanding of successful interventions for the Indian setting. Considering the diversity of Indian geography and culture, there is a need to adopt locally acceptable and feasible interventions. This systematic review was planned with the primary objective of collating available evidence on technologies that strengthen immunization across all age groups in India.

## Methods

### Search strategy and selection criteria

The protocol of this systematic review was registered in the Open Science Framework (OSF) registries database with a registration ID of osf.io/r42gm and can be accessed from their website (https://doi.org/10.17605/OSF.IO/R42GM).^19^ It is in accordance with the Preferred Reporting Items for Systematic Reviews and Meta-Analyses (PRISMA) reporting guidelines, and was started as a compendium of all evidence related to technologies affecting immunization in all geographical areas across the globe. This manuscript focuses on the evidence specific to the Indian context in the form of a systematic review as part of the Lancet Citizen’s Commission’s assignment on a collective effort towards generating evidence from India in the realm of health technology, human resources, governance, finance and citizens’ engagement for realizing universal health coverage (UHC) in India.

#### Database search

We searched three databases viz. PubMed, Cochrane Library and Google Scholar, through January 1, 2011 to July 31, 2021, to include evidence from the last decade. The search strategy was designed to cover evidence related to technologies and interventions that affect immunization coverage across all populations and regions of India. The term “*Technology*” was defined as ‘the application of scientific knowledge to practical purposes in any field’,^20^ which includes methods, techniques, and instrumentation. We used the Boolean operators ‘OR’ and ‘AND’ and combined four broad search blocks viz: 1) MeSH and Title abstract terms for ‘vaccination’ and ‘immunization’; 2) MeSH and Title abstract terms for ‘patient acceptance’ and ‘vaccine uptake’; and 3) MeSH and Title abstract terms for types of technologies, interventions and strategies. The details of PubMed search strategy are provided in Supplementary Table S1. The same search terms were used for Cochrane Library.

#### Study selection

All observational and experimental studies (randomized controlled trials, quasi-experimental studies, cohort studies, case-control studies or cross-sectional studies) from India, which assessed any kind of technology affecting immunization coverage, were included. Studies reporting impact of an intervention, even without a control arm (single-arm trials), were also included in the review in order to cover all possible technologies that play a role in improving immunization coverage. The included studies were conducted across all age groups and population types (pregnant women, caregivers, health care professionals, persons with co-morbid conditions, vulnerable populations). We excluded non-English articles, protocols, conference abstracts, narrative reviews, systematic reviews, qualitative studies, modelling studies, letters, correspondence, guidelines, multi-national studies when data from Indian population was unavailable, opinion pieces, commentaries, editorials, blogs and newspaper articles. Unpublished and grey literature sources were not assessed.

#### Data analysis

The primary outcomes of interest were technologies that affect immunization coverage. Articles retrieved from all three databases were imported into the DistillerSR software. Following removal of duplicates, titles and abstracts were screened for eligibility by two reviewers independently; discrepancies were resolved by mutual dialogue or by consultation with another reviewer. Full texts of eligible articles were retrieved and data extracted in DistillerSR, which included study identification details, study setting, place of study, duration, study design, number and types of vaccines studied, details of the number and types of technologies discussed. No authors were contacted for missing/additional information in the full text articles. The effect size(s) pertaining to the primary endpoint(s) of the study were extracted and presented, as reported in the publication. The findings from the adjusted analysis were presented, wherever available; no pooled analysis was conducted.

#### Quality assessment

The Mixed Methods Appraisal Tool (MMAT) 2018 was used to appraise the methodological quality of included studies independently by two reviewers in Microsoft Excel.^21^ Discrepancies in the scores were discussed and resolved, wherever applicable. The assessment included questions related to methodological approach, sampling procedure, response rate, confounders, measurement of outcomes and analysis. All eligible studies were included in the analysis regardless of their quality scores.

### Role of the funding source

The Lancet Commission had no role in study design, data collection, data analysis, data interpretation, or writing of the report.

## Results

### Study selection

**Fig. 1:**
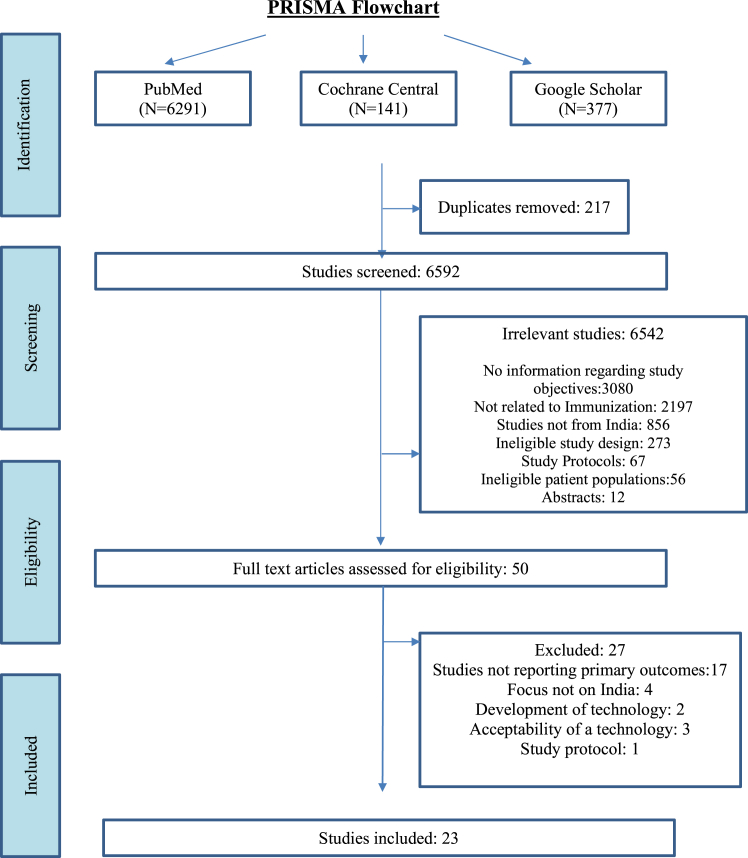
Study selection. PRISMA Flowchart showing selection and inclusion of the studies in the review.

The study selection details are shown in the PRISMA flow diagram (Fig. 1). The search identified 6809 articles. After removing duplicates (n = 217), 6592 articles were screened for titles and abstracts, of which 6542 articles were excluded. Full texts of 50 India-specific articles were obtained and assessed for eligibility. Twenty-three studies were included in this systematic review, after excluding 27 articles that did not report the primary outcomes including a modelling study which showed impact of community health workers on immunization coverage.^22^ The reasons for full-text exclusion are presented in Supplement S2.

### Study characteristics

**Fig. 2:**
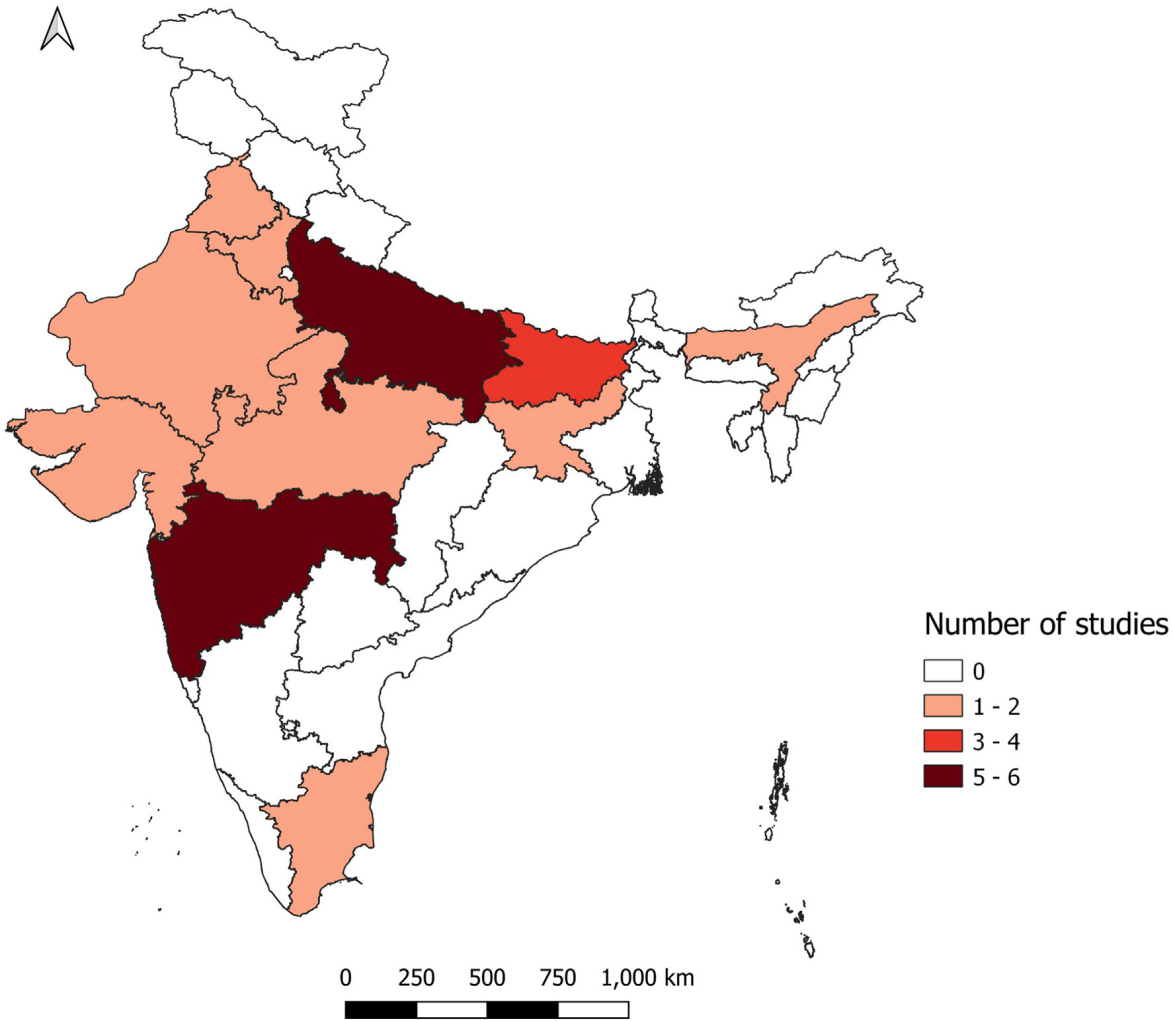
Map of India with states showing the location of the studies included in the review.

The study characteristics and the quality assessment scores of all the 23 included studies (with and without a comparison group) are described in Table 1, whereas those excluding the single-arm trials (17/23) are summarized in Supplementary Table S3. Majority (18/23; 78.26%) were published between 2016 and 2021. One study had pan-India data,^23^ while the rest were from the states of Assam (1),^24^ Bihar (3),^25^, ^26^, ^27^ Gujarat (1),^28^ Haryana (1),^29^ Jharkhand (2),^30^^,^^31^ Madhya Pradesh (1),^32^ Maharashtra (4),^33^, ^34^, ^35^, ^36^ Punjab (1),^37^ Rajasthan (2),^38^^,^^39^ Tamil Nadu (1)^40^ and Uttar Pradesh (5).^41^, ^42^, ^43^, ^44^, ^45^ Three studies were conducted in two or more states.^23^^,^^28^^,^^41^ Most (21/23, 91.3%) were community-based; one was school based and one was conducted in a health care centre. Eleven (47.8%) studies reported data from rural settings, six (26.1%) from urban/peri-urban settings, and six (26.1%) from both (rural and urban/peri-urban) settings. A map showing the distribution of study sites is presented in Fig. 2. Almost half of the studies (12/23) were from the Empowered action group (EAG) states (Bihar, Jharkhand, Rajasthan and Uttar Pradesh), which have high fertility, poor socio-demographic and health indicators. There was limited representation from the north-eastern and southern parts of the country. The most common study designs were quasi-experimental studies (10), randomised controlled trials (RCT) (6), cross-sectional studies (3), programme evaluation studies (using secondary data) (2) and mixed methods studies (2). Children were the commonest (19/23; 82.6%) target population, followed by pregnant women (4/23; 17.4%). Fifteen studies assessed multiple vaccines recommended for children, two polio, one measles, one MR, one hepatitis B, while two studied maternal tetanus toxoid, and one maternal influenza vaccine.

**Table 1:** Study characteristics and quality assessment using MMAT (n = 23).

S. No.	Author	Study design	Study setting	State	Study subjects	Sample size	Technology	Intervention	Control	Main findings	MMAT scoring
1	Chakraborty A et al. (2021)	Randomized Controlled Trial	Rural	Madhya Pradesh	Parents of Children	8204	Kilkari maternal messaging programme	Kilkari (automated voice) calls from the 12th week of pregnancy up until the child’s first birthday for immunization reminders and messaging for immunization benefits	No calls	Kilkari exposure was not associated with improvement of full and timely immunization coverage but it did increase timely immunization at birth. (Probit coefficient: 0.08, 95% CI 0.08–0.24).	••••○
2	Choudhary et al. (2021)	Quasi-experimental study	Rural and urban	Uttar Pradesh	Children eligible under Polio SIA	Variable across different rounds	Community level social mobilization	Social mobilization initiative (mobilisation through community workers and supplementary immunization activities)	Areas with no social mobilization initiative	The adjusted mean of outcome indicators was reported for each outcome. The mean booth coverage of intervention areas was 82.8 (95% CI 82.5–83.2), significantly higher (p < 0.001) by 36.4 percentage points than that of control areas [46.4% (95% CI 45.8–46.9)]. The intervention areas [66.3% (95% CI 65.7–66.9)] had a significantly higher (p < 0.001) conversion rate of ‘unvaccinated houses-to-vaccinated houses’ as compared to controls [54% (95% CI 53.2–54.7)]. Intervention areas had higher conversion rate of ‘Refusal houses-to-Acceptor houses’ [73.7% (95% CI 71.8–75.5)] as compared to control areas [65.5% (95% CI 63.6–67.3)] p < 0.01; there was a significantly lower (p < 0.05) rate of remaining ‘unvaccinated’ houses in intervention areas [4.9 (95% CI 4.8–5.1)] compared to non-intervention areas [5.9% (95% CI 5.8–6.0)]. The intervention areas had a significantly (p < 0.01) higher level of community engagement [89.0% (95% CI 88.9–89.2)], than non-intervention areas [70.8% (95% CI 70.6–71.1)].	•••○○
3	Summan et al. (2021)	Quasi-experimental study	Rural and urban	Select 260 districts from across India	Children	9674	Mission Indradhanush	Mission Indradhanush program	Districts with no Mission Indradhanush program	The Difference In Difference (DID) likelihood of receiving full immunization was 27% (95% confidence interval [CI]: 0.11–0.42, p < 0.01, Linear probability models (LPM)) higher among children under 2 years old residing in MI phase 1 and 2 districts (intervention group) as compared with those residing elsewhere (control group). The DID likelihood of children in the intervention groups was also 9% higher for OPV0 (CI: 0.02–0.15, p < 0.05, LPM), 9% higher for OPV1 (CI: 0.04–0.14, p < 0.01, LPM), 11% higher for OPV2 (CI: 0.02–0.19, p < 0.05, LPM), 16% higher for OPV3 (CI: 0.04–0.27, p < 0.01, LPM), 5% higher for BCG (CI: 0.01–0.09, p < 0.05, LPM), and 19% higher for hepatitis B birth dose (CI: 0.11–0.28, p < 0.01, LPM). The DID likelihood in phase 1&2 intervention group to have received age-appropriate vaccines as per recommended schedule was 8% higher (CI: 0.00–0.15, p < 0.05, LPM) than the control group.	•••••
4	Chen YJ et al. (2019)	Secondary data analysis	Rural	Gujarat & Maharashtra	Children	9580	Jyotigram Yojana (JGY)-rural electrification program	Jyotigram Yojana (JGY)-rural electrification program in Gujarat	Maharashtra with no JGY program	JGY increased the probability of children receiving critical vaccinations. The probit coefficient for BCG was 0.06 (95% CI 0.027–0.102, p < 0.01), for measles it was 0.122 (95% CI 0.057–0.187, p < 0.01), for DPT (all doses) 0.035 (95% CI −0.015 to 0.085) and for Polio (all doses) 0.036 (95% CI −0.005 to 0.077, p < 0.1). The probability of receiving all these vaccines increased significantly post-JGY implementation in Gujarat.	••••○
5	Giduthuri JG (2019)	Quasi-experimental study	Peri/Sub-Urban	Maharashtra	Clinicians providing ANC services	30	Sensitization and engagement of clinicians’ for recommending influenza vaccines to pregnant women	Physicians provided with Antenatal influenza vaccination (AIV) recommendations (global, academic and local) intended to motivate clinicians’ influenza vaccination practices for pregnant women coming for ANC. Note: Randomization of clinicians to an intervention and control arm was done separately for middle-class and slum sites.	Physicians not provided with any intervention	Estimated median rates of antenatal influenza immunization increased from 2.6% in Study Period (SP) 1–12.2% in SP2 (adj OR = 5.2, 95% CI 2.4–11.0) among middle-class active clinicians, but rates remained stable among middle-class controls (0.2% in SP1 and 0.1% in SP2). Among middle-class active clinicians, the median rate of taken opportunities for AIV strongly increased further from SP2 to SP3 (adj OR = 4.4, 95% CI 2.4–7.9). After the second interaction (SP3), middle-class active clinicians were vaccinating at a substantially higher rate of 37.8%, while the rate in middle-class control clinicians remained unchanged (0.2%).	•••○○
6	Murthy N et al. (2019)	Quasi-experimental study	Urban	Maharashtra	Pregnant women	2016	mMitra voice message	Women in the intervention group received mMitra voice messages two times per week throughout their pregnancy and until their infant turned 1 year of age	Pregnant women who did not receive mMitra voice messages	The intervention group performed significantly better on fully immunizing the infants (Adjusted OR 1.531, 95% CI 1.141–2.055, p = 0.005).	•••○○
7	Newtonraj A et al. (2019)	Cross-sectional analytic	Rural	Tamil Nadu	Children	420	Measles Rubella (MR) Vaccination Campaign in the rural area of Kanchipuram district, Tamilnadu	Measles Rubella (MR) Vaccination Campaign in the rural area of Kanchipuram district, Tamilnadu	No	Among the total sample of 420 children, 380 children (90.5% (range 87.4%–93.0%)) were found to be vaccinated and 40 children (9.5% (range 7.0%–12.6%)) were found to be unvaccinated immediately after phase 1 of the MR vaccine campaign	•••••
8	Vaidyanathan (2019)	Randomized Controlled Trial	Rural and Urban	Maharashtra	Children	2352	Information Education Communication (IEC) training through school children of adolescent age (Child to Child/Child to parent)	Standardized structured IEC strategy on immunization in addition to routine propaganda by government of India (GOI), media etc.	Routine propaganda by GOI, media, etc.	Age-appropriate full immunization coverage from birth to 5 years was 51% in rural and 67% in urban experimental groups before IEC, and it was 88% and 85% post-IEC in rural and in urban areas, respectively, KW = 13.5, p = 0.003. BCG to measles dropout rate was initially 22% in experimental and 17% in control groups that were found to be 11% and 17%, respectively, after IEC.	•••••
9	Powell-Jackson et al. (2018)	Randomized Controlled Trial	Rural	Uttar Pradesh	Mothers of children aged 0–36 months	722	Health education to mothers regarding tetanus and the benefits of DPT vaccine face-to-face through home visits	Mothers were randomly assigned in a ratio of 1:1:1 to 1 of 3 study arms: mothers in the first treatment group received information framed as a gain (e.g., the child is less likely to get tetanus and more likely to be healthy if vaccinated), mothers in the second treatment group received information framed in terms of a loss (e.g., the child is more likely to get tetanus and suffer ill health if not vaccinated)	The third arm acted as a control group, with no information given to the mother.	The proportion of children with DPT3 was 28% in the control group and 43% in the 2 groups receiving information, giving a difference of 14.6 percentage points (95% CI 7.3–21.9, p < 0.001). Children whose mothers received the information were 52% more likely to receive DPT3 than children in the control group. The information intervention increased the rate of measles vaccination by 22 percentage points (risk difference: 22%, 95% CI 14%–30%, p < 0.001; relative risk: 1.53, 95% CI 1.29–1.80) and the rate of full immunization by 14 percentage points (risk difference: 14%, 95% CI 8%–21%, p < 0.001; relative risk: 1.72, 95% CI 1.29–2.29).	••••○
10	Seth R et al. (2018)	Randomized Controlled Trial	Rural	Haryana	Children	608	Automated mobile phone reminders, with and without compliance linked incentives like mobile phone talk time	There were two intervention arms: automated mobile phone reminders alone, or automated reminders with compliance-linked incentives in the form of mobile phone talk time	No automated mobile phone reminders or incentives	Immunization coverage at enrolment and End of Study, Control: 33.3 (0–66.7) to 41.7 (23.1–69.2). Automated reminders: 33.3 (0–58.3) to 40.1 (30.8–69.2). Automated reminders with compliance-linked incentives: 33.3 (0–58.3) to 50.0 (30.8–76.9). Overall, 33.3 (0–58.3) to 43.8 (25.0–75.0). Children in the compliance-linked incentive group were significantly more likely to have received timely immunizations (40.8%; p < 0.03) compared with children in the control (31.3%) or automated mobile phone reminder groups (26.7%)	•••••
11	Choudhuri, G et al. (2017)	Quasi-experimental study	Urban	Uttar Pradesh	School children	11,250	Educational intervention to school children about hepatitis B	Screening of an educational documentary film on HBV in 430 intervention schools	6 non-intervention schools	The baseline HBV vaccination level among students receiving the intervention was 21%. Two years after the intervention, 45% of students (N = 4284) reported being vaccinated at intervention schools compared to 22% (N = 1264) at non-intervention schools.	•••○○
12	Ganguly E et al. (2017)	Cross-sectional analytic	Rural	Rajasthan	Children	5007	Rural Effective Affordable Comprehensive Health Care (REACH)-Village mapping by GPS, village household health information data recorded by community volunteers, computerized health data tracking to generate immunization due list	Rural Effective Affordable Comprehensive Health Care (REACH)-Village mapping by GPS, village household health information data recorded by community volunteers, computerized health data tracking to generate immunization due list utilizing government functionaries	No	About 14 months after initiation of the REACH strategy, full immunization coverage increased dramatically to 88.7%, partial immunization declined to 10.3%, and only 1.0% did not receive any immunization, compared with the results of the benchmark IIHMR survey (2008) to represent the pre-intervention rates. The coverage rates of individual vaccines were similar to the percentage of children fully immunized; 97.2% of the children had received BCG, 95.1% of the children had received 3 doses each of DPT and OPV, and immunization against measles had been received by 89.2% of children.	••••○
13	Haenssgen MJ et al. (2017)	Secondary data analysis	Rural and urban	Uttar Pradesh & Bihar	Children	54,852	Polio mass immunization high intensity campaign	Polio Mass Immunization Campaign.	No	Children in Bihar exhibit a ‘higher’ (4.3% greater odds of immunization) probability of vaccination uptake when exposed to higher polio campaign intensity. Conversely, high exposure is linked to ‘lower’ (‘decrease’ of 5.45% in the odds of a child to be fully immunized) attainment of full immunization in Uttar Pradesh.	•••••
14	More et al. (2017)	Randomized Controlled Trial	Peri/Sub-Urban	Maharashtra	Children	4544	“Community Resource Centre” delivered multiple interventions through community organizers educated about health through home visits, group meetings, day care, community events	20 clusters with Resource Centre offering 1 Microplanning 2 Emphasis on Communication. 3 Home Visits, Group Meetings, Day Care for Malnourished Children 4 Community Events 5 Counsellers	20 clusters without Community Resource Centre	The proportion of immunized children in the intervention and control group was similar in intention to treat (ITT) group (OR 1.30, 95% CI 0.84–2.01); but were greater in intervention group when assessed per protocol (OR 1.73, 95% CI 1.05–2.86)	•••○○
15	Nagar R. et al. (2017)	Randomized Controlled Trial	Rural	Rajasthan	Children	198	Digital NFC (Near Field Communication) pendant with and without voice call reminder system	Two intervention groups: Pendant Only: the immunization record was digitally stored on a pendant with black thread, worn by the child. Pendant + Voice Call Reminders: children received the pendant as described above and mothers received voice call reminders the day before and the day of the camp, along with a missed camp message for mothers who failed to attend.	NFC enabled sticker stuck on the immunization card.	Neither the NFC necklace nor the necklace with additional voice call reminders directly resulted in an increase in infant immunization timeliness through DTP3. DTP3 completion within two months from the time of registration was higher in the Pendant (37.7%) and Pendant and Voice arms (38.7%) compared to the Control (Sticker) arm (27.4%).	••••○
16	Prinja S et al. (2017)	Quasi-experimental study	Rural	Uttar Pradesh	Pregnant women	3201	m-Health application delivered through ASHA workers	Development and implementation of an m-health application used as a job-aid by ASHAs for registering pregnant women and for providing real-time guidance through key counselling points, decision support and simple referral algorithms for various maternal and child health issues and aid early identification, treatment and referral.	Blocks where mHealth application was not introduced	The coverage of maternal ≥2 tetanus toxoid vaccination increased in the intervention area by 4.28%. However, the change was not statistically significant.	•••••
17	Sengupta P et al. (2017)	Mixed methods study	Urban	Punjab	Children	647	Government funded community-based intervention-outreach clinic, community guardian	A government funded outreach vaccination programme for migrant communities living in slums.	Similar migrant slums with routine services	Uptake of routine vaccines administered in under 1 year of age was significantly (p ≤ 0.05) higher in the intervention clusters than the control. The likelihood of full immunization against 6 vaccine preventable diseases by the age of 1 year was more than twice than the control clusters [OR: 2.27 (95% CI 1.12–4.60); p = 0.023].	••••○
18	Balakrishnan R et al. (2016)	Quasi-experimental study	Rural	Bihar	Pregnant women	19,880	mHealth and community health worker training	An m-health platform used for case management by frontline community health workers. Pregnant women were registered and the child can be followed up till 6 years. Modules include pregnancy registration, birth preparedness, delivery, post-natal care, exclusive breastfeeding, immunization and growth charts.	Rest of Bihar with no mHealth intervention	Pregnant mothers received at least one TT vaccine 79.38% (95% CI 58.90–80.26) compared to 74.12% in the same district the previous year and 80% in the rest of Bihar in the same year.	•••○○
19	Jain M et al. (2015)	Mixed methods study	Rural	Jharkhand & Uttar Pradesh	Children		‘‘My Village Is My Home’’ (MVMH) tool (poster sized record of every infant in the community)	Large Poster Sized record consisting of a table on which every child in the community has a row depicting their immunization status.	No	The immunization coverage rates before and during the use of MVMH tool were available for Uttar Pradesh. In Uttar Pradesh there was an increase in the immunization rates for BCG from 82.3% to 88.5%. Similarly, there were small changes in OPV (54.2%–58.8%), DPT1 (83.6%–86.1%) and DPT3 (68.9%–72.1%), Measles immunization rates decreased from 71.4% to 67%.	•○○○○
20	Scobie HM et al. (2015)	Cross-sectional analytic	Rural and Urban	Jharkhand	Children	1018	Measles vaccination campaign	Government run phase 2 of a Measles Campaign.	No	MCV coverage among children aged 9 months to <10 years was 61.0% (95% CI 54.4%–67.7%). At the end of the campaign, 53.7% (95% CI 46.5%–60.9%) of children 12 months to <10 years of age received ≥2 MCV doses, while a large proportion of children remained under-vaccinated (1-dose) (34.0%, 95% CI 28.0%–40.0%) or unvaccinated (12.3%, 95% CI 9.3%–16.2%).	•••••
21	Goel S et al. (2012)	Quasi-experimental study	Rural and Urban	Bihar	Children		“Muskaan Ek Abhiyan (Smile)” Campaign (intersectoral coordination, awareness generation by women groups, budgetary support, monitoring and supervision mechanism, tracking beneficiaries, incentives to service providers) run by Government in Bihar	Review and strengthening of microplans Intersectoral Coordination between ICDS and Health Involvement of Mahila Mandals Performance based Incentives. Strengthening Monitoring and Evaluation. Enhanced Political Commitment.	Other EAG states.	The proportion of fully immunized 12–23-month-old children in Bihar increased significantly from 19% in 2005 to 49% in 2009 (p < 0.001). The coverage of BCG also increased significantly from 52.8% to 82.3% (p < 0.001), DPT-3 from 36.5% to 59.3% (p < 0.001), OPV-3 from 27.1% to 61.6% (p < 0.001) and measles from 28.4% to 58.2% (p < 0.001).	•••••
22	Pradhan N et al. (2012)	Quasi-experimental study	Urban	Bihar	Children		Urban immunization outreach, a multi-pronged strategy (increase in immunization site, plan logistics, community mobilization, supervision, vaccine drives etc.)	Within the framework of existing government drives—Increasing immunization sites, ensuring sufficient, staff for providing injections, planning required logistics, improving community mobilization, providing supervision, using reported data for action and supporting special complementary vaccination drives	No	With the outreach services, vaccination coverage increased from baseline by 121% for BCG, 121% for DPT 1, 148% for DPT-2, 133% for DPT-3, 122% for Measles, 120% for TT-1 and 170% for TT-2. The proportion of both children left out and not completing their DPT vaccination series decreased 47% and 35% respectively.	••••○
23	Ryman TK et al. (2011)	Quasi-experimental study	Rural	Assam	Children	800	Reaching Every District (RED) approach, a multi-pronged intervention (planning, outreach, community mobilization, supervision and monitoring)	3 districts received strengthening core sub-national routine vaccination program functions by re-establishing outreach services; providing supportive supervision; monitoring and using data for action; improving planning and resource management; and increasing community links with service delivery	3 comparison districts received no additional intervention except routine services. 8 districts received only training in RED approach but limited oversight.	During the intervention, coverage significantly increased in both Comprehensive-RED and comparison districts. Children at follow-up were 2.1 times (95% CI 1.5–3.0) more likely to be fully vaccinated compared with baseline in Comprehensive-RED districts, and 2.1 times (95% CI 1.6–2.8) more likely to be fully vaccinated at follow-up compared with baseline in comparison districts. In the 2 Comprehensive-RED districts the DTP1, DTP3, and measles coverage and the percentage of children who were fully vaccinated increased 8, 15, 20, and 18 percentage points, respectively. In comparison districts, coverage increased 16, 16, 20, and 17 percentage points for DTP1, DTP3, measles, and percentage of children fully vaccinated, respectively.	•••○○

### Quality assessment of studies

Though qualitative studies were not included, the qualitative component scoring was also provided for two mixed-methods studies as a requirement of MMAT. Almost all (22/23, 95.7%) studies had a consolidated score of three or more; one study had a score of one. No article was excluded from the analysis based on quality. A detailed assessment of the quality of each study using the MMAT is provided in Supplementary Table S4.

### Technologies for strengthening immunization coverage

**Fig. 3:**
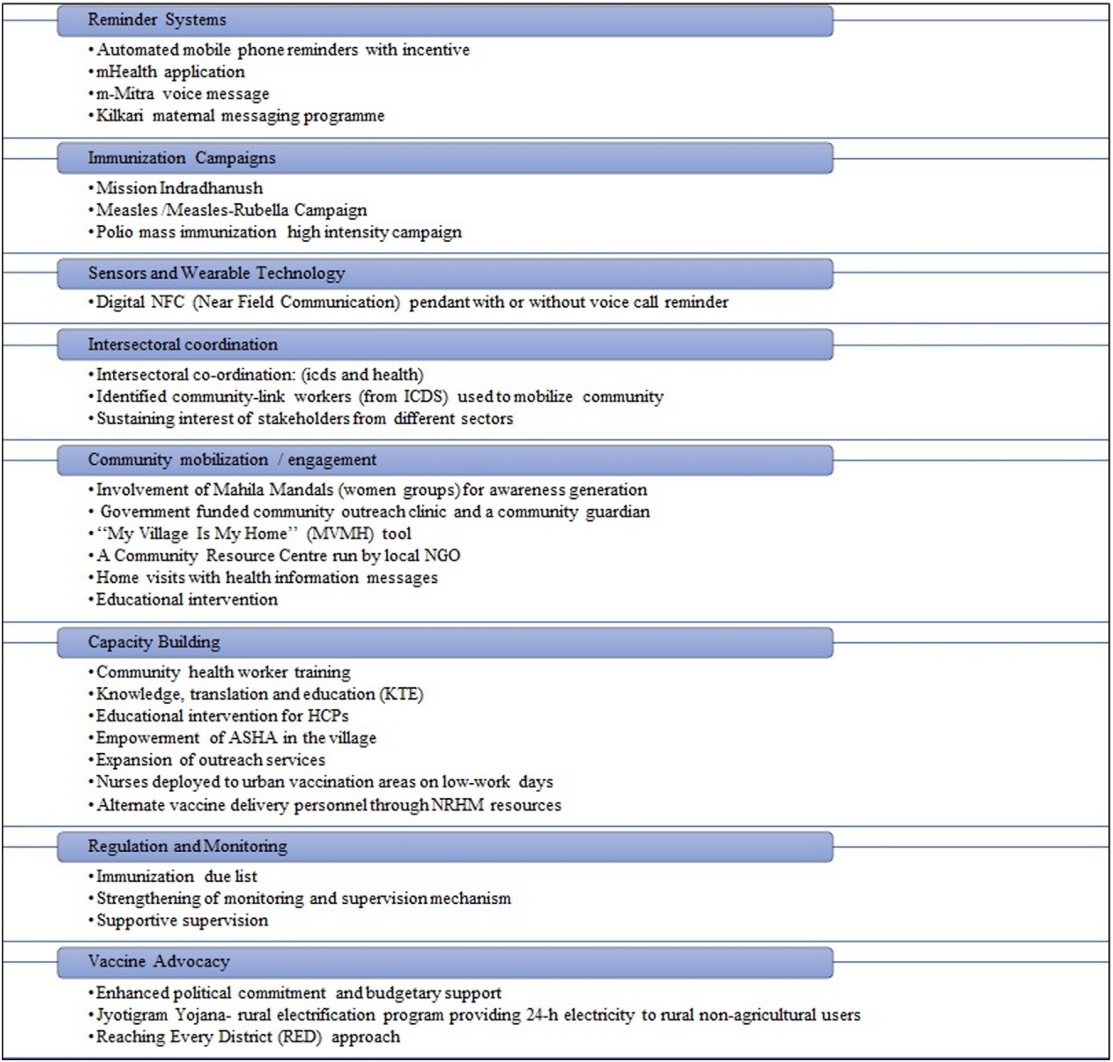
Technologies that affect immunization coverage across different settings in India.

The technologies identified in the studies were broadly categorised under eight different heads based on the type as shown in Fig. 3. These include the reminder systems, immunization campaigns, sensors and wearable technologies, intersectoral coordination, community mobilization/engagement, capacity building, regulation and monitoring and vaccine advocacy. Four studies reported technologies involving reminder systems which included automated mobile phone reminders with incentives,^29^ mHealth application,^27^ voice messages (mMitra)^33^ and the Kilkari messaging program.^32^ There was only one study that reported the use of sensors and wearable technology wherein a bead was attached to a thread and worn by the participant.^38^ This was the digital Near-Field Communication (NFC) pendant which was provided with or without a voice call reminder to the study participants. Five studies reported the use of immunization campaigns as technologies that improved coverage. These included assessment of the impact of national immunization programs or initiatives like the high-intensity Mission Indradhanush, the Measles-Rubella Campaign, the Polio mass immunization campaigns.^23^^,^^40^^,^^44^

Collaborative approaches such as intersectoral coordination were also studied, mostly in the context of the Integrated Child Development Scheme (ICDS), which is a government aided program for early childhood care and development and works by improving community engagement through Anganwadi Centres. Other community mobilisation-related interventions included the involvement of women groups, especially appointed community guardians and home visits by health personnel. Capacity building by training and empowerment of health workers and deployment of staff in increased numbers, were commonly instituted technologies. One study reported the effect of a rural electrification program and showed how a non-health technological intervention had an effect on immunization rates.^28^

The majority of the technologies showed improvements in coverage or timeliness. The “my village my home” (MVMH) campaign, a simple poster-sized community based tool to record and monitor the vaccination status of every child in the community by the community health workers was promising.^30^ Assessed in few districts of Jharkhand and Uttar Pradesh in India and Timor Leste, the MVMH tool improved immunization coverage and timeliness. An RCT from Haryana showed enhanced impact of compliance-linked incentive-phone talk time given with automated mobile phone reminders on timeliness compared to the control or automated mobile phone reminder groups.^29^ A computerised immunization due list as part of the Rural Effective Affordable Comprehensive Health Care (REACH) technology studied in rural Rajasthan was successful in improving coverage.^39^ This technology used village mapping by GPS and computerised health data tracking. The community level social mobilisation (CLSM) initiative involving mobilisation through community workers and supplementary immunization activities including fixed-booth and house-to-house polio immunization in Uttar Pradesh was unique in countering vaccine hesitancy during the post-polio endemic period.^44^ The ‘Muskaan Ek Abhiyan’ (the smile campaign) was an effective multisectoral strategy in Bihar of enhanced intersectoral coordination, awareness generation, increased budgetary support, monitoring and supervision, tracking of beneficiaries and performance-based incentives to service providers.^25^ Educational interventions to the school children^36^^,^^42^ and mothers of young children^43^ were also encouraging.

Some of the newer technologies did not improve coverage such as the digital NFC pendant^38^ and the comprehensive RED strategy (Reaching Every District),^24^ although they did lead to improvement in program quality. The impact of technologies also varied across states. For example, a high-intensity polio campaign resulted in a higher probability of vaccine uptake in Bihar while lower in Uttar Pradesh.^41^

## Discussion

The present review highlights the technologies that impact immunization coverage across all ages and populations in India. Twenty-three articles were included and more than half of them were quasi-experimental studies and RCTs. The technologies identified primarily included reminder systems, community mobilisation and capacity building related technologies. Almost all these technologies resulted in an increase in immunization coverage, however, the effectiveness results were variable for some, such as reminder systems; they also differed across states and settings.

Majority (82.6%) of the studies in the present review reported technologies for childhood immunization. The findings were similar to the recent global reviews for this population, wherein educational interventions, implementation of mandatory vaccination in schools, sending timely reminders, provider-directed interventions and financial incentives were found to improve childhood and adolescent vaccination coverage.^46^^,^^47^ Demand generation, modified vaccine delivery approaches, cash transfer programs, health systems strengthening and novel technology usage were also associated with increased immunization coverage of infants from low- and middle-income countries (LMICs).^48^ We did not find many studies on cash transfer programs and modified delivery approaches in the Indian context, which needs further exploration. Our review also included three studies on pregnant women, all of which assessed mHealth based technologies but showed variable results. Provision of pertussis vaccination by midwives at the place of antenatal care, automated reminders within the electronic medical record and increased provider awareness of recommendations have earlier been shown to be associated with higher maternal vaccine uptake.^49^

In the present review, we did not find much evidence on populations other than children and pregnant women. This depicts a significant lack of studies in the area of adult immunization, immunization of special/vulnerable populations and occupational immunization. The dearth of studies in these populations emphasises the need for a life-course approach to immunization, covering individuals as they progress through different stages of life viz. adolescence, adulthood and old age. There is also a lack of disease burden data on the adults and immunocompromised populations. These population groups are often deprived of vaccines due to lack of knowledge about vaccines among public and health care providers, lack of standard guidelines and protocols, and non-inclusion in the Universal Immunization Program (UIP). Integration and intersectoral coordination may, therefore, be necessary for vaccine access among these population groups.

The supply-side technologies identified in this review were mostly directed at capacity building in the forms of teaching and training and increasing the workforce for immunization. The primary processes studied were directed at increasing community engagement. Increasing workforce may not always be a feasible option, given the lack of funding and resources. The review also had limited representation from the north-eastern and southern parts of the country. In a country as vast and varied as India, differences in terrain, political interest, health care financing and population dynamics across states can result in certain technologies failing in certain settings. Except for one study,^35^ we did not find much evidence of technologies for sensitising healthcare providers about new vaccines, vaccine recommendations and immunization policies for different populations in the Indian context.

In this review, most of the technologies were well established interventions. A new technology identified was the Jyotigram Yojana (JGY): a rural electrification program that resulted in an increased uptake of critical childhood vaccines. A big challenge in LMICs, like India, where the ambient temperatures are high and the electricity supply is unreliable, is the maintenance of cold chain storage for vaccines,^50^ which can be improved through rural electrification. Interestingly, India has recently rolled out an electronic vaccine intelligence network (eVIN) system in some states in a phased manner. The eVIN system digitises the vaccine stock management, logistics and temperature tracking at all levels (national to sub-district) of vaccine storage, enabling the real-time monitoring of all the cold chain points. The MVMH-offline tool was found to be promising to track vaccination in communities and has been adapted by several state governments.^30^ The m-Health education programs of automated voice calls such as Kilkari and mMitra, have been scaled in many states across India, reaching millions of subscribers, suggesting the scalability of mobile based technologies in settings like India. Further, they also contribute to empower mothers. The digital NFC pendant was an innovative technology, although it did not improve the coverage significantly. The RED strategy was assessed in only one study in the present review. Developed in 2002 by WHO, it is a multifaceted strategy aimed at improving immunization services and includes re-establishing outreach services, supportive supervision, monitoring and data usage, improving resource management and increasing community service delivery links.^51^ It has been shown to strengthen the immunization programs in other countries,^52^ although further evaluation is needed in Indian settings.

Very few novel machine-based technologies were evaluated in India, as compared to other countries. The possible reasons could be more emphasis on strengthening of existing capacity building and community engagement efforts for improving immunization coverage, lack of funding to explore newer machine-based technologies and notable differences in effectiveness of a technology due to diversity in the Indian population.

The strengths of this review lies in the fact that we included all study designs and populations, to cover the whole spectrum of technologies. Additionally, the quality of most studies included was above-average. There are, however, certain limitations. First, this review did not include qualitative study designs. Second, our search was limited to the Indian context and it is possible that the inclusion of evidence from countries with settings similar to India could have provided evidence on newer technologies useful for the Indian context. Third, our search was limited to English language publications only; it is possible that studies in regional languages may have been missed. Nonetheless, most of the scientific research from India is published in English-language journals. Additionally, limiting our search to three databases may have resulted in missing certain studies. Fourth, there is a possibility of publication bias, with unpublished studies and those with negative results being missed. A bibliometric analysis of the relevant studies may be considered in the future, which may uncover emerging trends, identify research gaps and help derive novel ideas for further investigation.^53^

Multiple interventions may be necessary to improve the routine immunization systems in India, as evidenced from multipronged interventions in four studies.^25^^,^^26^^,^^34^^,^^39^ Improving vaccine access may be a key issue to improve vaccination coverage, especially in marginalized and hard-to-reach populations, for which additional human resources dedicated to social mobilization, advocacy, and community engagement will be crucial.^44^

For districts with the largest number of unimmunized children, the ones with the lowest coverage, better mapping and tracking tools for identifying beneficiaries and geospatial analysis may be useful. Vaccine mandates can be introduced for healthcare workers and occupationally-exposed employees. The UIP in India has introduced many vaccines for children, however, the platform needs to be further expanded to include adults, elderly and high-risk groups.^54^ Based on the recent success of Co-WIN portal for COVID-19 vaccination, India has initiated a pilot digital universal immunization program to keep the electronic immunization records of new-borns and pregnant mothers with features of reminders, online appointments, tracking on drop-outs and digital vaccination certificates. This is suggestive of newer technologies being adopted, based on local experience, acceptance and success. Future research on newer technologies is needed to improve vaccine coverage and timeliness, including cost-effectiveness estimates. Research and development for novel vaccine candidates, multivalent vaccines, and improved vaccine delivery systems (such as microneedle patches) to reduce dependence on needles and cold-chain should be considered.

This review of 23 studies identified several technologies that strengthen immunization programmes in India. Our findings will also benefit other similar countries in the South Asian region and LMICs. The technologies that improve programmatic and health system improvements and strategic planning, such as aiding in the preparation of the immunization-due list, capacity building efforts and engagement of community health workers were found to be effective in improving immunization coverage, although further evaluation in low-performing districts, different population groups, particularly vulnerable or vaccine-hesitant, and hard-to-reach areas are needed to ascertain their effectiveness across a range of settings. This will enable policy-makers to identify the most effective technologies for a diverse country like India. Our review had limited representation from populations other than children and pregnant women, which highlights the need for assessing the impact of established technologies in other population groups. Also, expanding the scope of this review to other LMICs, with settings like India, may help identify additional, potentially effective technologies. A multipronged strategy involving the most feasible, accessible, replicable and scalable technologies based on the local needs and perceived barriers, along-with planning and political will is needed to achieve the last mile immunization coverage in India.

## Contributors

GK conceived the study. ND and TK drafted the study protocol with inputs from MN, SA and RS. ND prepared the screening forms and data extraction sheet with input from all authors. TK, DV, SA, ND, CP and MN performed the article screening and data extraction. TK, CP, SA and MN performed the quality assessment. ND drafted the manuscript with inputs from TK and DV. All authors reviewed the manuscript and provided critical inputs. TK was involved in project administration, software management, coordination, communication, and led the whole review project. RS and GK provided guidance and supervision throughout the review process. We confirm that all authors had full access to all the data in the study and had final responsibility for the decision to submit for publication. ND, TK and DV directly accessed and verified the underlying data reported in the manuscript.

## Data sharing statement

No participant data was collected for systematic review.

## Editor note

The Lancet Group takes a neutral position with respect to territorial claims in published maps and institutional affiliations.

## Declaration of interests

We declare no competing interests.
