# Supplementary material for: Technologies for strengthening immunization coverage in India: a systematic review
Source: Lancet Reg Health Southeast Asia. 2023 Aug 8;23:100251. doi: 10.1016/j.lansea.2023.100251 (PMC10884965; doi:10.1016/j.lansea.2023.100251)
Supplement: Supplementary Tables S1–S4 [file mmc1.docx]

**Supplementary Appendix**

**Technologies for strengthening immunization coverage in India: A systematic review**

**Contents:**

Page 1: Supplementary Table S1. Detailed PubMed search strategy

Page 2 to 3: Supplementary Table S2. List of excluded studies at full-text screening

Page 4 to 13: Supplementary Table S3. Characteristics of studies with a comparison group, n=17

Page 14 to 15: Supplementary Table S4. Quality assessment of studies included in the review using the Mixed Methods Appraisal Tool (MMAT), 2018

| **Search Block** | **Search Terms** | **Results** |
| --- | --- | --- |
| 1 | immunization programs[MeSH Terms] OR vaccination coverage[MeSH Terms] | 16,280 |
| 2 | vaccin*[Text Word] OR immuniz*[Text Word] OR immunis*[Title/Abstract] OR Booster[Title/Abstract] | 5,19,437 |
| 3 | cover*[Title/Abstract] OR rate*[Title/Abstract] OR program*[Title/Abstract] OR Administration[Title/Abstract]  OR Drive[Title/Abstract] OR campaign*[Title/Abstract] OR strateg*[Title/Abstract] | 60,25,106 |
| 4 | #2 AND #3 | 1,56,278 |
| 5 | #1 OR #4 | 1,61,372 |
| 6 | patient acceptance of health care[MeSH Terms] OR vaccination refusal[MeSH Terms] | 1,65,642 |
| 7 | vaccin*[tiab] OR immuni*[tiab] | 6,02,043 |
| 8 | Strength*[Title/Abstract] OR Uptake[Title/Abstract] OR Hesitan*[Title/Abstract] OR Sust*[Title/Abstract] OR Refusal[Title/Abstract] OR Accept*[Title/Abstract] OR intent*[Title/Abstract] OR non-vaccination[Title/Abstract] OR anti-vax[Title/Abstract] OR Priorit*[Title/Abstract] OR Build*[Title/Abstract] OR Support*[Title/Abstract] OR Enhanc*[Title/Abstract] OR Extend[Title/Abstract] OR Establish*[Title/Abstract] OR Bolster[Title/Abstract] OR Challenge*[Title/Abstract] OR initiation[tiab] OR complet*[tiab] OR outreach[tiab] OR timeliness[tiab] OR improv*[tiab] OR Immunization / psychology | 93,94,950 |
| 9 | #7 AND #8 | 2,49,803 |
| 10 | #6 OR #9 | 4,10,677 |
| 11 | "technology"[MeSH Terms] OR "electronic health records"[MeSH Terms] OR "registries"[MeSH Terms] OR "health information systems"[MeSH Terms] OR "management information systems"[MeSH Terms] OR "internet"[MeSH Terms] OR "remote sensing technology"[MeSH Terms] OR "geographic information systems"[MeSH Terms] OR "satellite imagery"[MeSH Terms] OR "spatial analysis"[MeSH Terms] OR "wearable electronic devices"[MeSH Terms] OR "biosensing techniques"[MeSH Terms] | 8,07,741 |
| 12 | "technolog*"[Title/Abstract] OR "electronic health record*"[Title/Abstract] OR "health information system*"[Title/Abstract] OR "Registry"[Title/Abstract] OR "Telemedicine"[Title/Abstract] OR "remote sensing technolog*"[Title/Abstract] OR "vaccine tracking system*"[Title/Abstract] OR "geographic information system*"[Title/Abstract] OR "satellite imagery"[Title/Abstract] OR (("spatial analysis"[MeSH Terms] OR ("spatial"[All Fields] AND "analysis"[All Fields]) OR "spatial analysis"[All Fields]) AND "management information system*"[Title/Abstract]) OR "wearable electronic device*"[Title/Abstract] OR "pendant*"[Title/Abstract] OR "bracelet*"[Title/Abstract] OR "necklace*"[Title/Abstract] OR "bands"[Title/Abstract] OR "biosensing technique*"[Title/Abstract] | 8,23,710 |
| 13 | "reminder systems"[MeSH Terms] OR "telephone"[MeSH Terms] OR "answering services"[MeSH Terms] OR "cell phone"[MeSH Terms] OR "text messaging"[MeSH Terms] OR "smartphone"[MeSH Terms] OR "internet"[MeSH Terms] | 1,20,487 |
| 14 | "reminder*"[Title/Abstract] OR "text messag*"[Title/Abstract] OR "short message service"[Title/Abstract] OR "postcard*"[Title/Abstract] OR "computer*"[Title/Abstract] OR "poster*"[Title/Abstract] OR "messag*"[Title/Abstract] OR "mobile phone*"[Title/Abstract] OR "cell phone*"[Title/Abstract] OR "web based"[Title/Abstract] OR "social media"[Title/Abstract] OR "Instagram"[Title/Abstract] OR "Twitter"[Title/Abstract] OR "Facebook"[Title/Abstract] OR "mass media"[Title/Abstract] OR "radio*"[Title/Abstract] OR "TV"[Title/Abstract] OR "Television"[Title/Abstract] | 18,92,330 |
| 15 | #11 OR #12 OR #13 OR #14 | 32,18,663 |
| 16 | #5 AND #10 AND #15 | 10,085 |
| 17 | #16 AND 2011/01/01:2021/07/31[dp] AND English[la] NOT (letter[pt] OR comment[pt]) | 6,212 |
| 18 | ("technolog*"[Title/Abstract] OR "electronic health record*"[Title/Abstract] OR "health information system*"[Title/Abstract] OR "Registry"[Title/Abstract] OR "Telemedicine"[Title/Abstract] OR "remote sensing technolog*"[Title/Abstract] OR "vaccine tracking system*"[Title/Abstract] OR "geographic information system*"[Title/Abstract] OR "satellite imagery"[Title/Abstract] OR (("spatial analysis"[MeSH Terms] OR ("spatial"[All Fields] AND "analysis"[All Fields]) OR "spatial analysis"[All Fields]) AND "management information system*"[Title/Abstract]) OR "wearable electronic device*"[Title/Abstract] OR "pendant*"[Title/Abstract] OR "bracelet*"[Title/Abstract] OR "necklace*"[Title/Abstract] OR "bands"[Title/Abstract] OR "biosensing technique*"[Title/Abstract] OR intervention*[Title/Abstract]) AND (intervention[Title/Abstract]) | 6,89,191 |
| 19 | #11 OR #18 OR #13 OR #14 | 32,15,851 |
| 20 | #5 AND #10 AND #19 | 10,172 |
| 21 | #20 AND 2011/01/01:2021/07/31[dp] AND English[la] NOT (letter[pt] OR comment[pt]) | 6,291 |

Not reporting primary outcomes of impact of technology on immunization coverage

1. Posadzki, P., Mastellos, N., Ryan, R., Gunn, L. H., Felix, L. M., Pappas, Y., Gagnon, M. P., Julious, S. A., Xiang, L., Oldenburg, B., Car, J. Automated telephone communication systems for preventive healthcare and management of long-term conditions. *Cochrane Database Syst Rev.* 2016. 12:Cd009921 , 10.1002/14651858.CD009921.pub2
2. Oliveira, A., Selvaraj, K., Tripathy, J. P., Betodkar, U., Cacodcar, J., Wadkar, A. Kyasanur Forest Disease vaccination coverage and its perceived barriers in Goa, India-A mixed methods operational research. PLoS One. 2019. 14:e0226141 , 10.1371/journal.pone.0226141
3. Khan, J., Shil, A., Prakash, R. Exploring the spatial heterogeneity in different doses of vaccination coverage in India. PLoS One. 2018. 13:e0207209 , 10.1371/journal.pone.0207209
4. Choudhary, M., Solomon, R., Awale, J., Dey, R. Demand-side determinants of timely vaccination of oral polio vaccine in social mobilization network areas of CORE Group polio project in Uttar Pradesh, India. BMC Infect Dis. 2018. 18:222 , 10.1186/s12879-018-3129-2
5. Padmanabha, N., Kini, J. R., Alwani, A. A., Sardesai, A. Acceptability of human papillomavirus vaccination among medical students in Mangalore, India. Vaccine. 2019. 37:1174-1181, 10.1016/j.vaccine.2019.01.032
6. Som, M., Panda, B., Pati, S., Nallala, S., Anasuya, A., Chauhan, A. S., Sen, A. K., Zodpey, S. Effect of supportive supervision on routine immunization service delivery-a randomized post-test study in Odisha. Glob J Health Sci. 2014. 6:61-7 , 10.5539/gjhs.v6n6p61
7. Weiss, W. M., Choudhary, M., Solomon, R. Performance and determinants of routine immunization coverage within the context of intensive polio eradication activities in Uttar Pradesh, India: Social Mobilization Network (SM Net) and Core Group Polio Project (CGPP). BMC Int Health Hum Rights. 2013. 13:25, 10.1186/1472-698x-13-25
8. Choudhary, M., Solomon, R., Awale, J., Dey, R., Singh, J. P., Weiss, W. Significance of a social mobilization intervention for engaging communities in polio vaccination campaigns: Evidence from CORE Group Polio Project, Uttar Pradesh, India. J Glob Health. 2021. 11:07011 , 10.7189/jogh.11.07011
9. Kshatri, J. S., Palo, S. K., Panda, M., Swain, S., Sinha, R., Mahapatra, P., Pati, S. Reach, accessibility and acceptance of different communication channels for health promotion: a community-based analysis in Odisha, India. J Prev Med Hyg. 2021. 62:E455-e465 , 10.15167/2421-4248/jpmh2021.62.2.1929
10. Dave, K., Chinnakali, P., Thekkur, P., Desai, S., Vora, C., Desai, G. Attrition from Care and Clinical Outcomes in a Cohort of Sickle Cell Disease Patients in a Tribal Area of Western India. Trop Med Infect Dis. 2019. 4:#pages# , 10.3390/tropicalmed4040125
11. Gupta, M., Angeli, F., Bosma, H., Rana, M., Prinja, S., Kumar, R., van Schayck, O. C. Effectiveness of Multiple-Strategy Community Intervention in Reducing Geographical, Socioeconomic and Gender Based Inequalities in Maternal and Child Health Outcomes in Haryana, India. PLoS One. 2016. 11:e0150537 , 10.1371/journal.pone.0150537
12. Sebastian, J., Parthasarathi, G., Ravi, M. D. Impact of educational intervention on the best immunization practices among practicing health care professionals in a south Indian city. Ther Adv Vaccines Immunother. 2021. 9:25151355211032590 , 10.1177/25151355211032590
13. Johri, M., Chandra, D., Koné, G. K., Dudeja, S., Sylvestre, M. P., Sharma, J. K., Pahwa, S. Interventions to increase immunisation coverage among children 12-23 months of age in India through participatory learning and community engagement: pilot study for a cluster randomised trial. BMJ Open. 2015. 5:e007972 , 10.1136/bmjopen-2015-007972
14. Panda, B., Pati, S., Nallala, S., Chauhan, A. S., Anasuya, A., Som, M., Zodpey, S. How supportive supervision influences immunization session site practices: a quasi-experimental study in Odisha, India. Glob Health Action. 2015. 8:25772 , 10.3402/gha.v8.25772
15. Balasubramaniam, S., Kumar, S., Sethi, R., Charurat, E., Lalchandani, K., Schuster, A., Sood, B. Quasi-experimental Study of Systematic Screening for Family Planning Services among Postpartum Women Attending Village Health and Nutrition Days in Jharkhand, India. Int J Integr Care. 2018. 18:7 , 10.5334/ijic.3078
16. Johri, M., Chandra, D., Kone, K. G., Sylvestre, M. P., Mathur, A. K., Harper, S., Nandi, A. Social and Behavior Change Communication Interventions Delivered Face-to-Face and by a Mobile Phone to Strengthen Vaccination Uptake and Improve Child Health in Rural India: Randomized Pilot Study. JMIR Mhealth Uhealth. 2020. 8:e20356 , 10.2196/20356
17. Bettampadi D, Boulton ML, Power LE, Hutton DW. Are community health workers cost-effective for childhood vaccination in India? *Vaccine* 2019; **37**(22): 2942-51.

Primary focus not on India

1. Davis, R. Impact on child vaccination completion rates of short message services (SMS) reminders in developing countries. Pan Afr Med J. 2020. 35:12 , 10.11604/pamj.supp.2020.35.1.19442
2. Krimmel, T., Bannerji, R., Borysyuk, M., Schneider, S. M. Influenza Adherence Tool Kit: Implementation and Evaluation Among Allogeneic Hematopoietic Transplantation Recipients. Clin J Oncol Nurs. 2017. 21:339-344 , 10.1188/17.Cjon.339-344
3. Cassidy, B., Braxter, B., Charron-Prochownik, D., Schlenk, E. A. A quality improvement initiative to increase HPV vaccine rates using an educational and reminder strategy with parents of preteen girls. J Pediatr Health Care. 2014. 28:155-64 , 10.1016/j.pedhc.2013.01.002
4. Ofstead, C. L., Sherman, B. W., Wetzler, H. P., Dirlam Langlay, A. M., Mueller, N. J., Ward, J. M., Ritter, D. R., Poland, G. A. Effectiveness of worksite interventions to increase influenza vaccination rates among employees and families. J Occup Environ Med. 2013. 55:156-63 , 10.1097/JOM.0b013e3182717d13

Development of a technology

1. Katib, A., Rao, D., Rao, P., Williams, K., Grant, J. A prototype of a novel cell phone application for tracking the vaccination coverage of children in rural communities. 2015. 122:215-28 , 10.1016/j.cmpb.2015.08.008 Comput Methods Programs Biomed.
2. Giduthuri, J. G., Maire, N., Joseph, S., Kudale, A., Schaetti, C., Sundaram, N., Schindler, C., Weiss, M. G. Developing and validating a tablet version of an illness explanatory model interview for a public health survey in Pune, India. 2014. 9:e107374 , 10.1371/journal.pone.0107374 PLoS One.
3. Jarrett, S., Wilmansyah, T., Bramanti, Y., Alitamsar, H., Alamsyah, D., Krishnamurthy, K. R., Yang, L., Pagliusi, S. The role of manufacturers in the implementation of global traceability standards in the supply chain to combat vaccine counterfeiting and enhance safety monitoring. 2020. 38:8318-8325 , 10.1016/j.vaccine.2020.11.011 Vaccine.

Acceptability of a technology

1. Pérez, Myriam Cielo, Chandra, Dinesh, Koné, Georges, Singh, Rohit, Ridde, Valery, Sylvestre, Marie-Pierre, Seth, Aaditeshwar, Johri, Mira Implementation fidelity and acceptability of an intervention to improve vaccination uptake and child health in rural India: a mixed methods evaluation of a pilot cluster randomized controlled trial. Implementation science communications. 2020. 1:1-19 , #Doi#
2. Pérez, MC, Chandra, D, Koné, GK, Singh, R, Ridde, V, Sylvestre, MP, Seth, A . 3.3 Article 3 Implementation fidelity and acceptability of an intervention to improve vaccination uptake and child health in rural India: A mixed methods evaluation of a pilot cluster randomized controlled trial. 2020:253

Study protocol

1. Pramanik, S., Ghosh, A., Nanda, R. B., de Rouw, M., Forth, P., Albert, S. Impact evaluation of a community engagement intervention in improving childhood immunization coverage: a cluster randomized controlled trial in Assam, India. 2018. 18:534 , 10.1186/s12889-018-5458-x BMC Public Health.

| **S. No.** | **Author** | **Study Design** | **Study Setting** | **State** | **Study Subjects** | **Sample size** | **Technology** | **Intervention** | **Control** | **Main findings** | MMAT Scoring |
| --- | --- | --- | --- | --- | --- | --- | --- | --- | --- | --- | --- |
|  | Chakraborty A et al (2021) | Randomized Controlled Trial | Rural | Madhya Pradesh | Parents of Children | 8204 | Kilkari maternal messaging programme | Kilkari (automated voice) calls from the 12th week of pregnancy up until the child’s first birthday for immunization reminders and messaging for immunization benefits | No calls | Kilkari exposure was not associated with improvement of full and timely immunization coverage but it did increase timely immunization at birth. (Probit coefficient: 0.08, 95% CI 0.08-0.24). | ⚫⚫⚫⚫⭘ |
|  | Choudhary et al  (2021) | Quasi-experimental study | Rural and urban | Uttar Pradesh | Children eligible under Polio SIA | Variable across different rounds | Community level social mobilization | Social mobilization initiative (mobilisation through community workers and supplementary immunization activities) | Areas with no social mobilization initiative | The adjusted mean of outcome indicators was reported for each outcome. The mean booth coverage of intervention areas was 82.8 (95% CI 82.5-83.2), significantly higher (p < 0.001) by 36.4 percentage points than that of control areas [46.4% (95% CI 45.8-46.9)]. The intervention areas [66.3% (95% CI 65.7-66.9)] had a significantly higher (p < 0.001) conversion rate of ‘unvaccinated houses-to-vaccinated houses’ as compared to controls [54 % (95% CI 53.2-54.7)]. Intervention areas had higher conversion rate of ‘Refusal houses-to-Acceptor houses’ [73.7 % (95% CI 71.8-75.5)] as compared to control areas [65.5% (95% CI 63.6-67.3)] p<0.01; there was a significantly lower (p< 0.05) rate of remaining ‘unvaccinated’ houses in intervention areas [4.9 (95% CI 4.8-5.1] compared to non-intervention areas [5.9 % (95% CI 5.8 to 6.0)]. The intervention areas had a significantly (p < 0.01) higher level of community engagement [89.0% (95% CI 88.9-89.2)], than non-intervention areas [70.8% (95% CI 70.6-71.1]. | ⚫⚫⚫⭘⭘ |
|  | Summan et al (2021) | Quasi-experimental study | Rural and urban | Select 260 districts from across India | Children | 9674 | Mission Indradhanush | Mission Indradhanush program | Districts with no Mission Indradhanush program | The Difference in difference (DID) likelihood of receiving full immunization was 27% (95% confidence interval [CI]: 0.11–0.42, p < 0.01, Linear probability models (LPM)) higher among children under 2  years old residing in MI phase 1 and 2 districts (intervention group) as compared with those residing elsewhere (control group). The DID likelihood of children in the intervention groups was also 9% higher for OPV0 (CI: 0.02–0.15, p<0.05, LPM), 9% higher for OPV1 (CI: 0.04–0.14, p < 0.01, LPM), 11% higher for OPV2 (CI: 0.02–0.19, p < 0.05, LPM), 16% higher for OPV3 (CI: 0.04–0.27, p<0.01, LPM), 5% higher for BCG (CI: 0.01–0.09, p< 0.05, LPM), and 19% higher for hepatitis B birth dose (CI: 0.11–0.28, p < 0.01, LPM). The DID likelihood in phase 1&2 intervention group to have received age-appropriate vaccines as per recommended schedule was 8% higher (CI: 0.00–0.15, P<0.05, LPM) than the control  group. | ⚫⚫⚫⚫⚫ |
|  | Chen YJ et al (2019) | Secondary data analysis | Rural | Gujarat & Maharashtra | Children | 9580 | Jyotigram Yojana (JGY)- rural electrification program | Jyotigram Yojana (JGY)- rural electrification program in Gujarat | Maharashtra with no JGY program | JGY increased the probability of children receiving critical vaccinations. The probit coefficient for BCG was 0.06 (95% CI 0.027–0.102, p<0.01), for measles it was 0.122 (95%CI 0.057–0.187, p<0.01), for DPT (all doses) 0.035 (95% CI − 0.015–0.085) and for Polio (all doses) 0.036 (95% CI− 0.005–0.077, p<0.1). The probability of receiving all these vaccines increased significantly post-JGY implementation in Gujarat. | ⚫⚫⚫⚫⭘ |
|  | Giduthuri JG (2019) | Quasi-experimental study | Peri / Sub-Urban | Maharashtra | Clinicians providing ANC services | 30 | Sensitization and engagement of clinicians’ for recommending influenza vaccines to pregnant women | Physicians provided with Antenatal influenza vaccination (AIV) recommendations (global,  academic and local) intended to motivate clinicians’ influenza vaccination  practices for pregnant women coming for ANC.  Note: Randomization of clinicians to an intervention and control arm was done separately for middle-class and slum sites. | Physicians not provided with any intervention | Estimated median rates of antenatal influenza immunization increased from 2.6% in Study Period (SP)1 to 12.2% in SP 2 (adj OR = 5.2, 95% CI: 2.4–11.0) among middle-class active clinicians, but rates remained stable among middle-class controls (0.2% in SP1 and 0.1% in SP2). Among middle-class active clinicians, the median rate of taken opportunities for AIV strongly increased further from SP2 to SP3 (adj OR = 4.4, 95% CI 2.4–7.9). After the second interaction (SP3), middle-class active clinicians were vaccinating at a substantially higher rate of 37.8%, while the rate in middle-class control clinicians remained unchanged (0.2%). | ⚫⚫⚫⭘⭘ |
|  | Murthy N et al  (2019) | Quasi-experimental study | Urban | Maharashtra | Pregnant women | 2016 | mMitra voice message | Women in the intervention group received mMitra voice messages two times per week throughout their pregnancy and until their infant turned 1 year of age | Pregnant women who did not receive mMitra voice messages | The intervention group performed significantly better on fully immunizing the infants (Adjusted OR 1.531, 95% CI 1.141–2.055, p=0.005). | ⚫⚫⚫⭘⭘ |
|  | Vaidyanathan (2019) | Randomized Controlled Trial | Rural and Urban | Maharashtra | Children | 2352 | Information Education Communication (IEC) training through school children of adolescent age (Child to Child / Child to parent) | Standardized structured IEC strategy on immunization in addition to routine propaganda by government of India (GOI), media etc. | Routine propaganda by GOI, media, etc. | Age‑appropriate full immunization coverage from birth to 5 years was 51% in rural and 67% in urban experimental groups before IEC, and it was 88% and 85% post‑IEC in rural and in urban areas, respectively, KW=13.5, p=0.003. BCG to measles dropout rate was initially 22% in experimental and 17% in control groups that were found to be 11% and 17%, respectively, after IEC. | ⚫⚫⚫⚫⚫ |
|  | Powell-Jackson et al  (2018) | Randomized Controlled Trial | Rural | Uttar Pradesh | Mothers of children aged 0-36 months | 722 | Health education to mothers regarding tetanus and the benefits of DPT vaccine face-to-face through home visits | Mothers were randomly assigned in a ratio of 1:1:1 to 1 of 3 study arms: mothers in the first treatment group received information framed as a gain (e.g., the child is less likely to  get tetanus and more likely to be healthy if vaccinated), mothers in the second treatment  group received information framed in terms of a loss (e.g., the child is more likely to get tetanus and suffer ill health if not vaccinated) | The third arm acted as a control group, with no information given to the mother. | The proportion of children with DPT3 was 28% in the control group and 43% in the 2 groups receiving information, giving a difference of 14.6 percentage points (95% CI: 7.3- 21.9, p < 0.001). Children whose mothers received the information were 52% more likely to receive DPT3 than children in the control group. The information intervention increased the rate of measles vaccination by 22 percentage points (risk difference: 22%, 95% CI: 14%-30%, p < 0.001; relative risk: 1.53, 95% CI: 1.29-1.80) and the rate of full immunization by 14 percentage points (risk difference: 14%, 95% CI: 8%-21%, p < 0.001; relative risk: 1.72, 95% CI: 1.29-2.29). | ⚫⚫⚫⚫⭘ |
|  | Seth R et al  (2018) | Randomized Controlled Trial | Rural | Haryana | Children | 608 | Automated mobile phone reminders, with and without compliance linked incentives like mobile phone talk time | There were two intervention arms:  automated mobile phone reminders alone, or automated reminders with compliance-linked incentives in the form of mobile phone talk time | No automated mobile phone reminders or incentives | Immunization coverage at enrolment and End of Study, Control: 33.3 (0–66.7) to 41.7 (23.1–69.2). Automated reminders: 33.3 (0–58.3) to 40.1 (30.8–69.2). Automated reminders with compliance-linked incentives: 33.3 (0–58.3) to 50.0 (30.8–76.9). Overall, 33.3 (0–58.3) to 43.8 (25.0–75.0). Children in the compliance-linked incentive group were significantly more likely to have received timely immunizations (40.8%; P < .03) compared with children in the control (31.3%) or automated mobile phone reminder groups (26.7%) | ⚫⚫⚫⚫⚫ |
|  | Choudhuri, G et al  (2017) | Quasi-experimental study | Urban | Uttar Pradesh | School children | 11250 | Educational intervention to school children about hepatitis B | Screening of an educational documentary film on HBV in 430 intervention schools | 6 non-intervention schools | The baseline HBV vaccination level among students receiving the intervention was 21%. Two years after the intervention, 45% of students (N = 4284) reported being vaccinated at intervention schools compared to 22% (N = 1264) at non-intervention schools. | ⚫⚫⚫⭘⭘ |
|  | More et al  (2017) | Randomized Controlled Trial | Peri / Sub-Urban | Maharashtra | Children | 4544 | “Community Resource Centre” delivered multiple interventions through community organizers educated about health through home visits, group meetings, day care, community events) | 20 clusters with Resource Centre offering   1. Microplanning 2. Emphasis on Communication. 3. Home Visits, Group Meetings, Day Care for Malnourished Children, 4. Community Events 5. Counsellers | 20 clusters without Community Resource Centre | The proportion of immunized children in the intervention and control group was similar in intention to treat (ITT) group (OR-1·30, 95% CI 0·84–2·01); but were greater in intervention group when assessed per protocol (OR 1·73, 95% CI 1·05–2·86) | ⚫⚫⚫⭘⭘ |
|  | Nagar R. et al  (2017) | Randomized Controlled Trial | Rural | Rajasthan | Children | 198 | Digital NFC (Near Field Communication) pendant with and without voice call reminder system | Two intervention groups:  Pendant Only: the immunization  record was digitally stored on a pendant with black thread, worn by the child.  Pendant + Voice Call Reminders: children  received the pendant as described above and mothers received voice call reminders the day before and the day of the camp, along  with a missed camp message for mothers who failed to attend. | NFC enabled sticker stuck on the immunization card. | Neither the NFC necklace nor the necklace with additional voice call reminders directly resulted in an increase in infant immunization timeliness through DTP3. DTP3 completion within two months from the time of registration was higher in the Pendant (37.7%) and Pendant and Voice arms (38.7%) compared to the Control (Sticker) arm (27.4%). | ⚫⚫⚫⚫⭘ |
|  | Prinja S et al  (2017) | Quasi-experimental study | Rural | Uttar Pradesh | Pregnant women | 3201 | m-Health application delivered through ASHA workers | Development and implementation of an m-health application used as a job-aid by ASHAs for registering pregnant women and for providing real-time guidance through key counselling points, decision support and simple referral algorithms for various maternal and child health issues and aid early identification, treatment and referral. | Blocks where mHealth application was not introduced | The coverage of maternal ≥2 tetanus toxoid vacccination increased in the intervention area by 4.28%. However, the change was not statistically significant. | ⚫⚫⚫⚫⚫ |
|  | Sengupta P et al  (2017) | Mixed methods study | Urban | Punjab | Children | 647 | Government funded community-based intervention-outreach clinic, community guardian | A government funded outreach vaccination programme for migrant communities living in slums. | Similar migrant slums with routine services | Uptake of routine vaccines administered in under 1 year of age was significantly (p=<0.05) higher in the intervention clusters than the control. The likelihood of full immunization against 6 vaccine preventable diseases by the age of 1 year was more than twice than the control clusters [OR: 2.27 (95% CI: 1.12–4.60); p = 0.023]. | ⚫⚫⚫⚫⭘ |
|  | Balakrishnan R et al  (2016) | Quasi-experimental study | Rural | Bihar | Pregnant women | 19880 | mHealth and community health worker training | An m-health platform used for case management by frontline community health workers. Pregnant women were registered and the child can be followed up till 6 years. Modules include pregnancy registration, birth preparedness, delivery, post-natal care, exclusive breastfeeding, immunization and growth charts. | Rest of Bihar with no mHealth intervention | Pregnant mothers received at least one TT vaccine 79.38% (95% CI:58.90–80.26) compared to 74.12% in the same district the previous year and 80% in the rest of Bihar in the same year. | ⚫⚫⚫⭘⭘ |
|  | Goel S et al  (2012) | Quasi-experimental study | Rural and Urban | Bihar | Children |  | “Muskaan Ek Abhiyan (Smile)” Campaign (intersectoral coordination, awareness generation by women groups, budgetary support, monitoring and supervision mechanism, tracking beneficiaries, incentives to service providers) run by Government in Bihar | Review and strengthening of microplans,  Intersectoral Coordination between ICDS and Health Involvement of Mahila MandalsPerformance based Incentives. Strengthening Monitoring and Evaluation. Enhanced Political Commitment. | Other EAG states. | The proportion of fully immunized 12–23-month-old children in Bihar increased significantly from 19% in 2005 to 49% in 2009(P<0.001). The coverage of BCG also increased significantly from 52.8% to 82.3%(P<0.001), DPT-3 from 36.5 to 59.3%(P<0.001), OPV-3 from 27.1% to 61.6% (P<0.001) and measles from 28.4 to 58.2% (P<0.001). | ⚫⚫⚫⚫⚫ |
|  | Ryman TK et al (2011) | Quasi-experimental study | Rural | Assam | Children | 800 | Reaching Every District (RED) approach, a multi-pronged intervention (planning, outreach, community mobilization, supervision and monitoring) | 3 districts received strengthening core sub-national routine vaccination program functions by re-establishing outreach services; providing supportive supervision; monitoring and using data for action; improving planning and resource management; and increasing community links with service delivery | 3 comparison districts received no additional intervention except routine services.  8 districts received only training in RED approach but limited oversight. | During the intervention, coverage significantly increased in both Comprehensive-RED and comparison districts. Children at follow-up were 2.1 times (95% CI: 1.5–3.0) more likely to be fully vaccinated compared with baseline in Comprehensive-RED districts, and 2.1 times (95% CI: 1.6–2.8) more likely to be fully vaccinated at follow-up compared with baseline in comparison districts. In the 2 Comprehensive-RED districts the DTP1, DTP3, and measles coverage and the percentage of children who were fully vaccinated increased 8, 15, 20, and 18 percentage points, respectively. In comparison districts, coverage increased 16, 16, 20, and 17 percentage points for DTP1, DTP3, measles, and percentage of children fully vaccinated, respectively. | ⚫⚫⚫⭘⭘ |

|  | |  | | 1. Qualitative | | | | | 2. Randomized Controlled Trial | | | | | 3. Quantitative Non-randomized | | | | | 4. Quantitative Descriptive | | | | | 5. Mixed methods | | | | | MMAT Scoring |
| --- | --- | --- | --- | --- | --- | --- | --- | --- | --- | --- | --- | --- | --- | --- | --- | --- | --- | --- | --- | --- | --- | --- | --- | --- | --- | --- | --- | --- | --- |
| S. No. | **Author** | **S1** | **S2** | **1.1** | **1.2** | **1.3** | **1.4** | **1.5** | **2.1** | **2.2** | **2.3** | **2.4** | **2.5** | **3.1** | **3.2** | **3.3** | **3.4** | **3.5** | **4.1** | **4.2** | **4.3** | **4.4** | **4.5** | **5.1** | **5.2** | **5.3** | **5.4** | **5.5** |  |
|  | Chakraborty et al (2021) | Y | Y |  |  |  |  |  |  |  |  |  |  |  |  |  |  |  | Y | Y | Y | N | Y |  |  |  |  |  | ⚫⚫⚫⚫⭘ |
|  | Choudhary et al (2021) | Y | Y |  |  |  |  |  |  |  |  |  |  | N | Y | N | Y | Y |  |  |  |  |  |  |  |  |  |  | ⚫⚫⚫⭘⭘ |
|  | Chen et al (2019) | Y | Y |  |  |  |  |  |  |  |  |  |  | Y | Y | Y | N | Y |  |  |  |  |  |  |  |  |  |  | ⚫⚫⚫⚫⭘ |
|  | Giduthuri et al (2019) | Y | Y |  |  |  |  |  |  |  |  |  |  | N | Y | Y | N | Y |  |  |  |  |  |  |  |  |  |  | ⚫⚫⭘⭘⭘ |
|  | Murthy et al (2019) | Y | Y |  |  |  |  |  |  |  |  |  |  | Y | Y | N | N | Y |  |  |  |  |  |  |  |  |  |  | ⚫⚫⚫⭘⭘ |
|  | Newtonraj et al (2019) | Y | Y |  |  |  |  |  |  |  |  |  |  |  |  |  |  |  | Y | Y | Y | Y | Y |  |  |  |  |  | ⚫⚫⚫⚫⚫ |
|  | Vaidyanathan (2019) | Y | Y |  |  |  |  |  | Y | Y | Y | Y | Y |  |  |  |  |  |  |  |  |  |  |  |  |  |  |  | ⚫⚫⚫⚫⚫ |
|  | Powell-Jackson et al (2018) | Y | Y |  |  |  |  |  | Y | Y | Y | Y | C |  |  |  |  |  |  |  |  |  |  |  |  |  |  |  | ⚫⚫⚫⚫⭘ |
|  | Seth et al (2018) | Y | Y |  |  |  |  |  | Y | Y | Y | Y | Y |  |  |  |  |  |  |  |  |  |  |  |  |  |  |  | ⚫⚫⚫⚫⚫ |
|  | Choudhuri G et al (2017) | Y | Y |  |  |  |  |  |  |  |  |  |  | Y | Y | C | N | Y |  |  |  |  |  |  |  |  |  |  | ⚫⚫⚫⭘⭘ |
|  | Ganguly et al (2017) | Y | Y |  |  |  |  |  |  |  |  |  |  | Y | Y | Y | N | Y |  |  |  |  |  |  |  |  |  |  | ⚫⚫⚫⚫⭘ |
|  | Haenssgen (2017) | Y | Y |  |  |  |  |  |  |  |  |  |  | Y | Y | Y | Y | Y |  |  |  |  |  |  |  |  |  |  | ⚫⚫⚫⚫⚫ |
|  | More et al (2017) | Y | Y |  |  |  |  |  | Y | Y | Y | N | N |  |  |  |  |  |  |  |  |  |  |  |  |  |  |  | ⚫⚫⚫⭘⭘ |
|  | Nagar et al (2017) | Y | Y |  |  |  |  |  | Y | Y | Y | N | Y |  |  |  |  |  |  |  |  |  |  |  |  |  |  |  | ⚫⚫⚫⚫⭘ |
|  | Prinja et al (2017) | Y | Y |  |  |  |  |  |  |  |  |  |  | Y | Y | Y | Y | Y |  |  |  |  |  |  |  |  |  |  | ⚫⚫⚫⚫⚫ |
|  | Sengupta et al (2017) | Y | Y | Y | Y | Y | Y | Y |  |  |  |  |  | Y | Y | Y | N | Y |  |  |  |  |  | Y | Y | Y | Y | C | ⚫⚫⚫⚫⭘ |
|  | Balakrishnan et al (2016) | Y | Y |  |  |  |  |  |  |  |  |  |  | Y | Y | C | N | Y |  |  |  |  |  |  |  |  |  |  | ⚫⚫⚫⭘⭘ |
|  | Jain et al (2015) | Y | Y | Y | N | N | N | C |  |  |  |  |  | Y | N | Y | N | Y |  |  |  |  |  | N | N | N | Y | N | ⚫⭘⭘⭘⭘ |
|  | Scobie H et al (2015) | Y | Y |  |  |  |  |  |  |  |  |  |  |  |  |  |  |  | Y | Y | Y | Y | Y |  |  |  |  |  | ⚫⚫⚫⚫⚫ |
|  | Goel et al (2012) | Y | Y |  |  |  |  |  |  |  |  |  |  | Y | Y | Y | Y | Y |  |  |  |  |  |  |  |  |  |  | ⚫⚫⚫⚫⚫ |
|  | Pradhan et al (2012) | Y | Y |  |  |  |  |  |  |  |  |  |  | Y | Y | Y | N | Y |  |  |  |  |  |  |  |  |  |  | ⚫⚫⚫⚫⭘ |
|  | Ryman et al (2011) | Y | Y |  |  |  |  |  |  |  |  |  |  | Y | Y | Y | N | N |  |  |  |  |  |  |  |  |  |  | ⚫⚫⚫⭘⭘ |
|  | Summan et al (2011) | Y | Y |  |  |  |  |  |  |  |  |  |  | Y | Y | Y | Y | Y |  |  |  |  |  |  |  |  |  |  | ⚫⚫⚫⚫⚫ |
